# Cloning, expression, purification and characterization of human mitochondrial carbonic anhydrase VA

**DOI:** 10.1007/s13205-015-0334-1

**Published:** 2016-01-07

**Authors:** Danish Idrees, Sudhir Kumar, Syed Abdul Arif Rehman, Samudrala Gourinath, Asimul Islam, Faizan Ahmad, Md. Imtaiyaz Hassan

**Affiliations:** 1 Centre for Interdisciplinary Research in Basic Sciences, Jamia Millia Islamia, Jamia Nagar, New Delhi, 110025 India; 2 School of Life Sciences, Jawaharlal Nehru University, New Delhi, 110025 India

**Keywords:** Carbonic anhydrase VA, Protein expression, Gluconeogenesis, Enzyme assay, Circular dichroism, Protein purification

## Abstract

Carbonic anhydrase VA (CAVA) is a mitochondrial enzyme that catalyzes the reversible hydration of CO_2_ to produce HCO_3_^−^ and proton. CAV is primarily involved in several biosynthetic processes such as ureagenesis, gluconeogenesis and lipogenesis by providing bicarbonate ion. Here, we report a new strategy for cloning, expression and purification for CAVA in the bacterial system followed by its biophysical characterization. The cDNA of CAVA, a 801 nucleotide long that encodes a 267-amino acid polypeptide of molecular mass of 30-kDa (excluding signal peptide), was sub-cloned in the expression vector pET21c and transformed into *Escherichia coli* strain BL21 (DE3) for expression. The recombinant protein was purified in two steps by Ni–NTA and DEAE weak anion-exchange chromatography under native condition from the supernatant, while inclusion bodies (IBs) were used to get protein under the denatured condition with a relatively high yield. CAVA was purified under denatured conditions in a single step using Ni–NTA chromatography. SDS-PAGE showed a band of 30-kDa, which was further confirmed as CAVA by Western blot and MALDI-TOF/MS. We further performed enzyme activity to ensure that both forms of purified proteins are enzymatically active. Measurements of secondary structure of the native, denatured and renatured proteins were carried out using circular dichroism. The purified protein can be further used for structural and biochemical studies.

## Introduction

Carbonic anhydrase V (CAV) is a mitochondrial enzyme (Dodgson et al. 1980) that catalyzes the hydration of carbon dioxide to produce proton and bicarbonate. CAV exists in two closely related mitochondrial isoform known as carbonic anhydrase VA (CAVA) and CAVB which have a distinct tissue distribution (Imtaiyaz Hassan et al. 2013). CAVB is expressed in the heart, skeletal muscle, kidney, salivary gland and spinal cord (Ghandour et al. 2000), while CAVA is localized in the mitochondria and expressed primarily in the liver (Saarnio et al. 1999). CAVA is actively involved in several biosynthetic processes such as ureagenesis, gluconeogenesis and lipogenesis and helps in glucose (Dodgson and Cherian 1989; Parkkila et al. 1998), fatty acid and urea synthesis (van Karnebeek et al. 2014). CAV is the only mammalian CA which is compartmentalized in a cell organelle and it has tyrosine at 64^th^ position instead of histidine, a catalytic proton shuttle residue which transfers proton from zinc-bound water to buffer (Boriack-Sjodin et al. 1995; Vitale et al. 2007). However, the proton shuttle transport in CAV is still not clear.

CAVA gene was localized to chromosome 16q24.3 and an unprocessed pseudogene has been assigned to 16p12 (Nagao et al. 1995). CAVA gene is composed of 915 nucleotides encoding a 305-amino acid protein with a molecular mass of 35-kDa. It has a hydrophobic N-terminal mitochondrial signal sequence of 38 amino acid residues and a catalytic domain of 267-amino acids. Based on the effect of CA inhibitors on these processes, a physiological role of CAVA in the biosynthetic processes has been suggested (Liang et al. 1993; Heck et al. 1994). In fact, mitochondria require CAV for these metabolic processes, and deregulation of this enzyme causes obesity (Arechederra et al. 2013), which is one of the most common human diseases, because of alteration in the metabolic substrate flux (Picard et al. 2000). Inhibition of CAVA has been identified as a novel anti-obesity strategy, over the other because of their serious cardiovascular or central nervous system (CNS) side effects. Several studies have provided insight that inhibition of CAVA with sulfonamide/sulfamate drug has potential as anti-obesity (Kaul and Ritschel 1988). However, better inhibitors are needed to overcome the problem of selectivity, since CAVA share a close homology, with other CA isoform, to design selective inhibitors of CAVA is challenging (Iqbal et al. 2015; Swenson 2014).

We tried to express the full length of CAVA in the bacterial system, but protein was unable to get solubilized. To overcome this problem, we have sub-cloned cDNA of CAVA into expression vector pET21c, encoding a 267-amino acid protein corresponding to the catalytic domain only (801 nucleotides and 30-kDa). Moreover, we have purified CAVA from both soluble supernatant and inclusion bodies (IBs). We further optimized the purification procedure to get the soluble proteins from inclusion bodies which have similar structure and enzyme activity in comparison to the protein purified from the supernatant. The aim of the present study is to construct a prokaryotic expression system for producing recombinant protein which can be used for structure–function studies in future.

## Materials and methods

### Strains and plasmid

Clone of CAVA was purchased from PlasmID Harvard Medical School. DH5α and BL21 (DE3) strains of *E. coli* were used for cloning and expression of the protein, respectively. *E. coli* cells harboring recombinant plasmid were grown aerobically at 37 °C in Luria–Bertani (Merck, Darmstadt, Germany) broth with 100 μg/ml ampicillin (Sigma, Saint Louis, MO, USA). Plasmid pET-21c (Novagen, WI, USA) was used as expression vector. Plasmid isolation, restriction enzyme digestion, ligation and competent cell preparation were carried out using standard procedure.

### Cloning of CAVA gene

The 801-bp coding region (39-305aa) was amplified by PCR from plasmid pENTR2231 containing the CAVA gene using forward and reverse primers 5′CTAGCTAGCTGTGCATGGCAAACCAGCAA3′ and 5′CGGCTCGAGTAAGGACCTTTGCCCTCATTAG3′, respectively. This amplification generated *Nhe1* and *Xho1* sites on each end of the amplified fragment and excluded the coding region (1-38aa) for the putative signal sequence. The CAVA gene was sub-cloned into pET21c vector with the C-terminal His6 tag.

### Expression of recombinant protein CAVA

The constructed expression vector (pET21c-CAVA) containing coding region of CAVA gene was transformed into *E. coli* BL21 (DE3) host cells by following the standard protocol. Overnight culture of the expression cells from a freshly transformed plate was inoculated in LB media, containing 100 μg/µl ampicillin, and incubated at 37 °C, with constant agitation at 220 rpm in an incubator shaker until the absorbance reaches 0.6 at 600 nm. The culture was induced by 0.50 mM IPTG (Sigma, Saint Louis, USA) and incubated for next 15 h at 16 °C. Cells were centrifuged at 7000*g* for 10 min at 4 °C. Then, cells were dissolved in 50 mM Tris–HCl buffer, pH 8.0 containing 500 mM NaCl, 5 % (v/v) glycerol, 5 mM β-mercaptoethanol, 0.1 mg/ml lysozyme, 100 mM phenyl methane sulfonyl fluoride (PMSF) and 1 % (v/v) triton X-100 (U. S. Biochemical Corp). Cell lysate was sonicated on ice and centrifuged for 30 min at 13,000 rpm at 4 °C. Supernatant was collected for purification of CAVA by the nickel affinity chromatography.

### Purification of CAVA under native condition

A clear supernatant was passed through Ni–NTA column which was pre-equilibrated with a buffer (50 mM Tris–HCl, pH 8.0, 500 mM NaCl, 5 % (v/v glycerol, 5 mM β-mercaptoethanol, 10 mM imidazole). After binding of protein, column was washed with 50 ml of washing buffer (50 mM Tris–HCl, pH 8.0, 500 mM NaCl, 5 % (v/v) glycerol, 5 mM β-mercaptoethanol, 20 mM imidazole) at 4 °C. Bound protein was eluted with 300 mM imidazole. The fractions were concentrated using Amicon Ultra 10 K device (Merck Darmstadt, Germany) and dialyzed against 50 mM Tris–HCl pH 8.0 buffer. The dialyzed sample was further loaded on Hi Trap DEAE FF (1 ml, 7 mm × 25 mm) column (GE healthcare) pre-equilibrated with 50 mM Tris–HCl buffer, pH 8.0. Bound proteins were eluted with increasing concentration of NaCl (0–1 M NaCl) in the 50 mM Tris–HCl pH 8.0 buffer. Elution was controlled by Akta purifier (GE healthcare) with a flow rate of 0.5 ml/min. CAVA eluted at 0.50 M NaCl was pooled, concentrated and stored for further study. The purity of protein was confirmed by sodium dodecyl sulfate-polyacrylamide gel electrophoresis (SDS-PAGE).

### Alternative methods of CAVA purification

We also report an alternative method of CAVA purification as a major portion of CAVA which was expressed as insoluble protein in the form of inclusion bodies (IBs). Earlier, we successfully purified the protein from the soluble form, but its yield was very low. To increase the yield of CAVA protein, we have tried a number of different methods to isolate the protein from IBs.

### Preparation and solubilization of IBs

*Escherichia coli* cells were centrifuged, pellets were resuspended in lysis buffer (50 mM phosphate buffer and 300 mM NaCl, pH 7.0) and lysed through sonicate, and IBs in the cell lysate were pelleted by low-speed centrifugation. Then, the pellet was again resuspended and washed with milli-Q water through centrifugation thrice to remove contaminations and got purified IBs. Solubilization of IBs is a vital step to obtaining the maximal amount of desired protein. We tried various denaturing conditions, such as urea, 1 % SDS, guanidinium chloride (GdmCl) and 1 % *N*-lauroylsarcosine to obtain maximum amount of our protein. CAVA was solubilized in all conditions. We solubilized the purified IBs with 50 mM phosphate buffer pH 7.0, 300 mM NaCl and 1 % *N*-lauroylsarcosine, and incubated on rocker for 30 min. We centrifuged the solubilized IBs for 30 min at 12,000 rpm and collected the supernatant.

### Purification of recombinant CAVA from IBs

Ni–NTA column was equilibrated with equilibration buffer (50 mM phosphate buffer pH 7.0, 50 mM NaCl and 1 % *N*-lauroylsarcosine). The collected supernatant was loaded on equilibrated Ni–NTA column. Then, the column was washed with washing buffer (50 mM phosphate buffer pH 7.0, 50 mM NaCl and 1 % *N*-lauroylsarcosine and 20 mM imidazole). The desired protein was eluted with elution buffer (50 mM phosphate buffer pH 7.0, 50 mM NaCl and 0.5 % *N*-lauroylsarcosine and 300 mM imidazole). The fractions of eluted CAVA protein were collected and checked for homogeneity on SDS-PAGE.

### Western blotting

Band of CAVA protein on SDS-PAGE was transferred onto the nitrocellulose membrane using standard procedure. Nitrocellulose membrane was blocked with 5 % non-fatty acid milk powder in phosphate-buffered saline (PBS) for 2 h followed by three washing. Conjugate Anti-His–HRP antibody (Penta-His, Qiagen) was incubated with the membranes for another 2 h and subsequently washed to remove the unbound antibody. Blot was developed by incubating the membrane for 2 min with 3, 30-diaminobenzidine (DAB) reagents (Zhongshan Biotech, Beijing) and 200 μl hydrogen peroxide.

### Mass spectrometry

To know the amino acid sequence of purified protein, we performed matrix-assisted laser desorption/ionization time of flight mass spectrometry (MALDI-ToF/MS). The band of CAVA from the SDS-PAGE was excised for tryptic digestion and subjected to mass spectrometer (Kratos Analytical, Shimadzu Group Company, Japan). We used standard procedure for the sequencing of CAVA, which was described elsewhere in detail (Hassan et al. 2007a, 2008). The peptide mass finger prints were used to identify protein by searching the SWISS-PROT and NCBI-nr databases using the Mascot 2.0 search engine with fragment mass tolerance of ±0.5 Da and max missed cleavages of one (Table 1).

**Table 1:** List of peptide fragments obtained after trypsinization

Start–end	Observed	Nominal mass (expected)	Nominal mass (calculated)	ppm	Peptide
38–63	2865.4580	2864.4507	2864.4028	17	R. SCAWQTSNNTLHPLWT
64–72	1141.6107	1140.6034	1140.6040	−0	R.QSPINIQWR.D
73–84	1430.7755	1430.7755	1429.7682	8	R.DSVYDPQLKPLR.V
169–185	1715.0111	1714.0038	1713.9665	22	K.EAVVGENGLAVIGVFLK
186–195	1160.6216	1159.6143	1159.6210	−6	K.LGAHHQTLQR.L
250–263	1529.8103	1528.8030	1528.7885	9	K.EPVEVAPSQLSAFR.T
276–290	1876.9592	1875.9519	1875.9270	13	K.MMVNNYRPLQPLMNR
291–304	1594.8141	1593.8068	1593.7899	11	R.KVWASFQATNEGTR.S

### Enzyme activity

To confirm the active nature of the purified protein, we performed enzyme activity of the native and renatured CAVA molecules. The *p*-nitrophenyl acetate (NPA) was used as the substrate to measure the activity of CAVA by recording the increase of absorbance at 400 nm (Ikai et al. 1978). The reaction cuvette contained 0.1 mM *p*-nitrophenyl acetate in 50 mM Tris pH 8.0, 5 % acetonitrile and enzyme in the concentration of 0.3 mg/ml for both CAII and CAVA at 25 °C. We have taken CAII as a positive control for activity. Esterase activity was carried out by following the release of nitrophenol which was measured at 400 nm by spectrophotometer (Jasco V-660, Model B 028661152) equipped with Peltier-type temperature controller (ETCS-761). All spectral measurements were done at least three times at 25 ± 0.1 °C.

### Circular dichroism

Secondary structure of CAVA was measured using circular dichroism (CD) in Jasco spectropolarimeter (model J-1500) equipped with Peltier type temperature controller. The far-UV CD spectra (250–200 nm) were recorded at 25 ± 0.1 °C using a cuvette of 1 mm path length cell and protein concentration of 0.3 mg/ml (10 μM). Samples of the native and renatured CAVA were prepared in 50 mM Tris–HCl and 50 mM phosphate buffer containing 150 mM NaCl at pH 8.0 and 7.0, respectively. Denatured sample was prepared in phosphate buffer with 1 % *N*-lauroylsarcosine at pH 7.0. The raw CD data were converted to the mean residue ellipticity, [*θ*] (degrees cm^2^ dmol^−1^), using the relation:

1 $$[ {\theta = {\text{Mo}}} [\theta ]/10lc$$

where [*θ*] is the observed ellipticity in millidegrees, Mo is the mean residue weight of the protein, *c* is the concentration in mg/ml, and *l* is the path length of the cell in centimetre. The CD spectra were uploaded onto the K2D2 server to estimate the secondary structural content of CAVA (Perez-Iratxeta and Andrade-Navarro 2008). This server is based on the comparison of real and predicted values, by means of the Pearson correlation coefficient (*r*) and the root mean square deviation (RMSD) of proteins with known three-dimensional structure. A detail of algorithm is described elsewhere (Johnson 1999; McPhie 2008; Sreerama and Woody 2000).

## Results

### Cloning and expression of CAVA

**Fig. 1:**
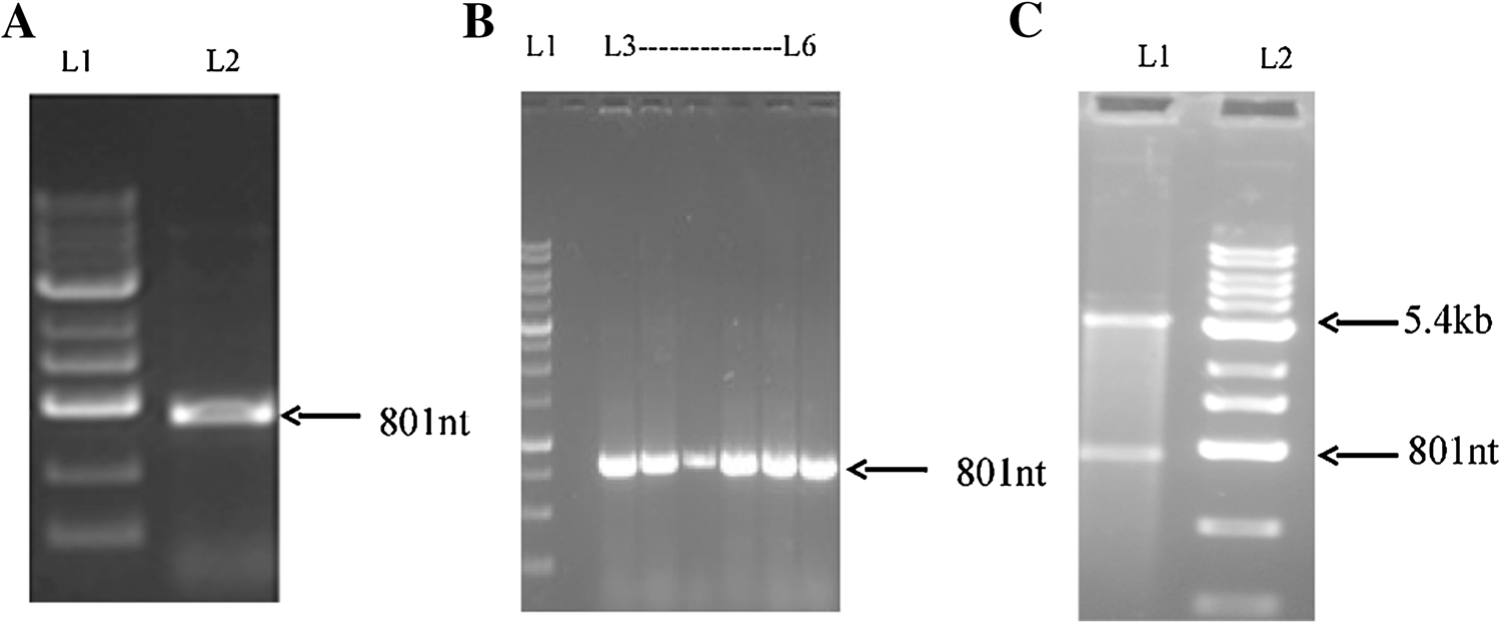
Cloning of CAVA gene: **a** amplified CAVA gene.* Lane 1*: Marker and *Lane 2* is an amplified product of 801 bp. **b** Confirmed constructed plasmid by colony PCR.* Lane 1* is a marker and lanes 3 to 8 are amplified products, further confirmed by restriction digestion. **c** Digested products back bone of 5.4 kb of pET21c and gene of CAVA of 801 nucleotides (*Lane 2*) and* Lane 1* is a marker

**Fig. 2:**
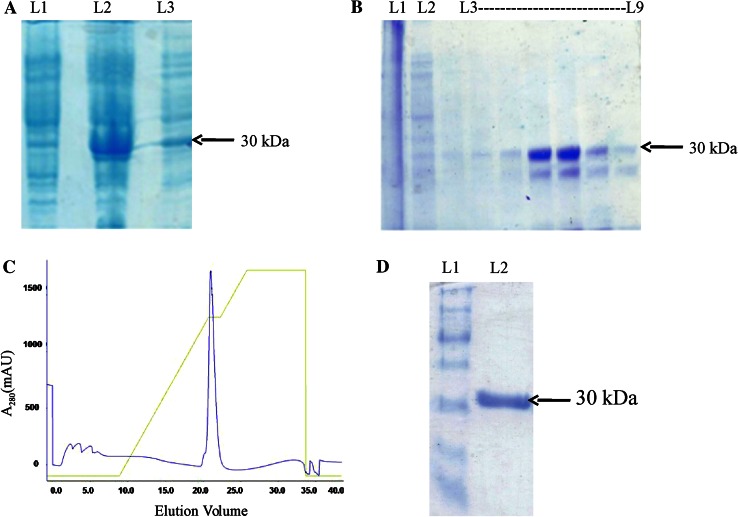
Purification of CAVA under native condition. **a** SDS-PAGE showing the expression of recombinant CAVA protein. **b** SDS-PAGE showing an eluent obtained from Ni–NTA column chromatography.* Lane 1* is flow through,* lane 2* is washing with 20 mM imidazole and* lanes 3 to 9* are eluent fractions. **c** Elution profile of ion-exchange chromatography. **d** SDS-PAGE of purified CAVA.* Lane 1* is a molecular mass marker and* lane 2* shows purified CAVA

The CAVA gene from plasmid pENTR223 was amplified by PCR, with *Nhe1* and *Xho1* site at the 5′ and 3′ end, respectively. The amplified product was 801 bp long (Fig. 1a). It was digested with same restriction enzymes *Nhe1* and *Xho1*, and subsequently ligated to the pET21c *Nhe1* and *Xho1* backbone fragment. This clone was confirmed by colony PCR (Fig. 1b) and restriction digestion with *Nhe1* and *Xho1* endonucleases (Fig. 1c). Constructed plasmid was further verified by DNA sequencing (not shown). The confirmed plasmid constructed pET21c-CAVA was transformed into *E. coli* BL21 (DE3). Recombinant protein was expressed at 16 °C by inducing with 0.5 mM IPTG. Overexpression of CAVA was clearly indicated on SDS-PAGE with an apparent molecular weight of 30-kDa (Fig. 2a).

### Purification of CAVA under native condition

To purify proteins in biologically active form from bacterial system, require an optimization of expression, solubilization of IBs and refolding experiments. Here, we expressed CAVA in *E*. coli BL21 (DE3). The major portion of the protein was expressed as insoluble fractions. Hence, we have purified CAVA successfully under the native as well as denatured conditions. The recombinant CAVA protein was isolated and purified to homogeneity at 4 °C from cell extract under the native condition. CAVA was found in the soluble bacterial extract, and obtained after the centrifugation of sonicated *E. coli* cell at 20,000*g*. CAVA was purified to homogeneity by two chromatographic steps on Ni–NTA affinity chromatography and DEAE ion-exchange chromatography. The eluted fractions after Ni–NTA affinity chromatography showed a significant level of contamination by other proteins (Fig. 2b). Eluted fractions were pooled, extensively dialyzed and loaded on to the DEAE anion exchanger column to get pure CAVA. Elution profile of DEAE anion exchanger column chromatography is shown in Fig. 2c. Fractions of protein obtained in DEAE anion exchanger column were subjected to SDS-PAGE to check the purity. A clear single band was observed on SDS-PAGE indicating the purity of CAVA (Fig. 2d). A summary of CAVA purification procedure is provided in Table 2.

**Table 2:** Purification summary

S. no.	Purification step	Volume (ml)	Protein (mg/ml)^b^	Total protein (mg)	Yield (100 %)
1	*E. coli* cell pellet^a^	60	43.91	2635.02	100
2	Supernatant	56	1.428	79.968	3.034
3	Ni-affinity chromatography	15	0.136	2.04	0.077
4	Weak anion chromatography (DEAE column)	2	0.504	1.008	0.038

^a^From 8 g of wet weight *E. coli* cell pellet (from 4 litre of batch)

^b^Protein concentration determined by Lowry assay using BSA as a standard protein

### Purification of CAVA under denatured condition

**Fig. 3:**
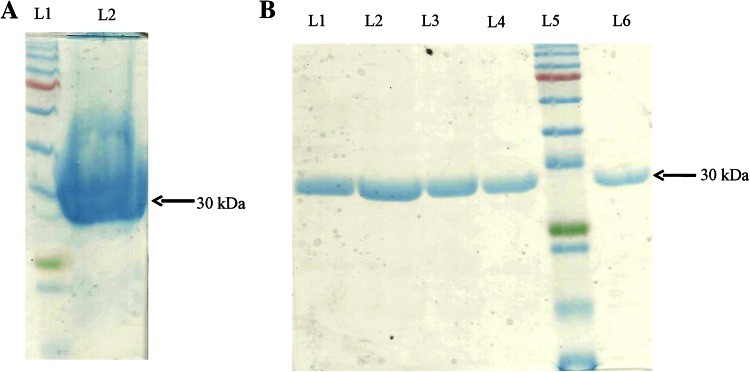
Purification of CAVA under denatured condition. **a** SDS-PAGE showing expression of CAVA in IBs.* Lane 1* shows a marker and* lane 2* is for CAVA, **b** SDS-PAGE shows an eluent obtained from Ni–NTA column chromatography (*lanes 1–4 * and *6*) and* lane 5* is marker (prestained protein marker, broad range (7–175 kDa)

To improve protein yield, we purified CAVA protein under denatured condition from IBs because large portion of recombinant CAVA protein was expressed there. We isolated IBs from cell extract and purified by sequential washing with milli-Q water to remove contaminants, especially non-specific proteins and proteases. Purified IBs of 30 kDa were checked on SDS-PAGE (Fig. 3a). We used 1 % *N*-lauroylsarcosine to solubilize the IBs among other denaturants because of problems in downstream processes. Solubilized IBs were loaded on an Ni–NTA affinity chromatography and purified in single chromatography step. The purity and yield of fractions of purified CAVA were checked on SDS-PAGE (Fig. 3b).

### Refolding of CAVA

Refolding of the protein is an important process to remove excess denaturants and provide appropriate condition for protein to spontaneously refold into correct conformation. There is no standard protocol for refolding of proteins. We refolded CAVA by dialyzing the solution against 50 mM phosphate buffer pH 7.0 and 150 mM NaCl for 24 h at 4 °C. Dialyzed protein was centrifuged to remove any precipitate formed during refolding. To further check that refolded protein is enzymatically active, we performed an enzyme assay. CAVA catalyzes the hydrolysis of *p*-nitrophenyl-acetate to *p*-nitrophenol, measured by recording the increase in absorbance with time. A significant increase in the optical density of solution at 400 nm clearly suggests that purified native and renatured CAVA is enzymatically active (data not shown).

### Sequencing and identification

**Fig. 4:**
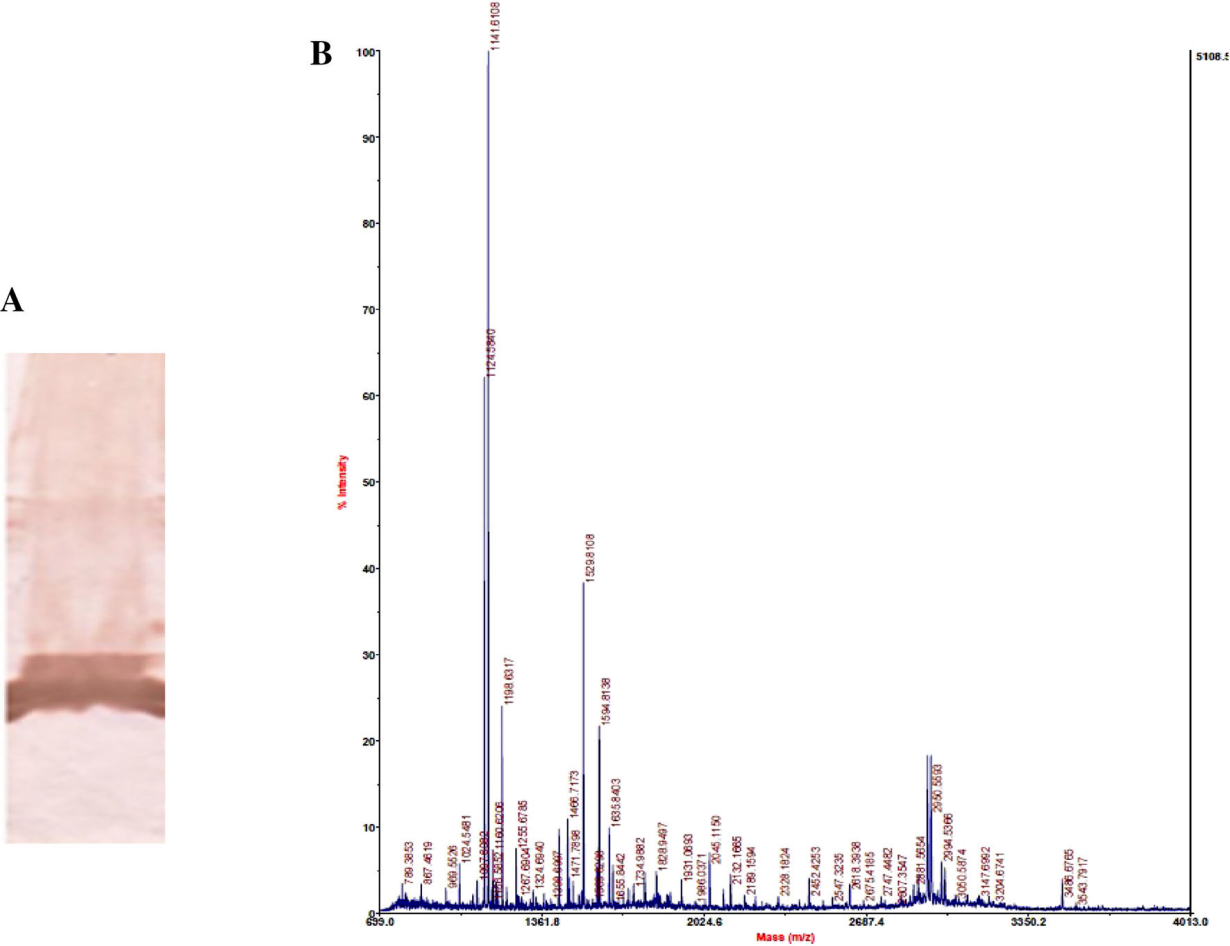
**a** Western blot analysis confirmed the presence of recombinant CAVA protein. **b** Peptide mass fingerprint of CAVA

The CAVA was confirmed by Western blot (Fig. 4a) and mass spectrometry MALDI-TOF (Fig. 4b). Mass spectrometry analysis identified many fragments of CAVA, clearly indicating this protein as human mitochondrial CAVA (Table 1). Both these studies clearly indicate that purified protein is CAVA.

### Secondary structure measurement

**Fig. 5:**
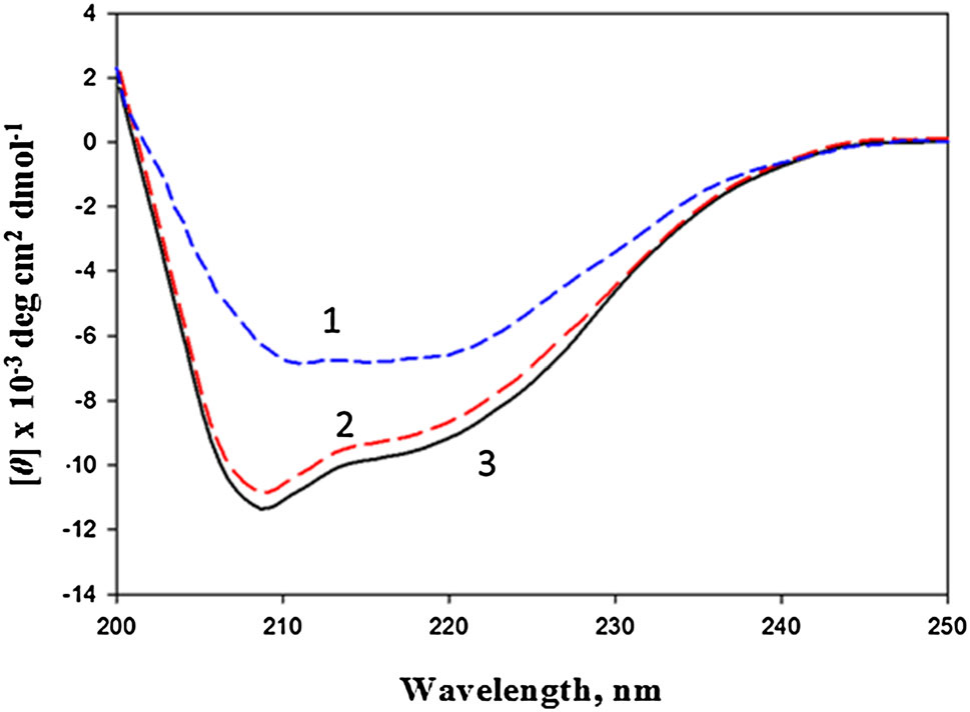
Far-UV CD spectra of recombinant protein CAVA. *Curves 1*, *2* and *3* represent spectra of denatured, renatured and native proteins, respectively

To estimate secondary structure contents, we performed the far-UV CD of denatured, renatured and native CAVA proteins at 25 °C in the region of 250 to 200 nm (Fig. 5). Spectra were analyzed to determine the secondary structure content of the protein with available programme (Perez-Iratxeta and Andrade-Navarro 2008). We used K2D2 server and found the secondary structural contents of the native CAVA as 20.2 % α-helix, 29.1 % β-sheet and 50.7 % random coil, for the renatured CAVA as 21.7 % α-helix, 27.2 % β-sheet and 51.3 % random coil, and for the denatured CAVA contents as 15.3 % α- helix, 21.7 % β-sheet and 63 % random coil. The crystal structure of human CAVA is not determined so far. However, we calculated the secondary structure content through secondary structure prediction server and homology modeling and a considerable correlation was observed between the theoretical calculation of secondary structure and calculation of the CD data. Moreover, refolding study clearly indicates that CAVA is a reversibly denatured protein.

## Discussion

We have successfully cloned, expressed and purified human CAVA (CAVA), which lacks the first 38 amino acid N-terminal residues for putative mitochondrial signal sequence. This is the first report of human CAVA protein purified in the bacterial system in both native and denatured conditions. However, mouse mitochondrial CAVB was successfully expressed and purified from bacterial system, but truncated form of CAVB (CAVB) could not be purified from the bacterial system due to its toxicity for host (Heck et al. 1994). We compared human CAVA with mouse CAVB, because it has 85 % homology and will try to find out the reason why truncated form of human CAVA is not toxic. We purified CAVA using the combination of Ni–NTA affinity and anion exchange chromatography used to purify recombinant protein yielded a pure and homogeneous protein but yield is very low under native conditions. We also used alternate methods to improve yield, prepared IBs and isolated CAVA from it under denaturing conditions. We successfully solubilized IBs using 1 % *N*-lauroylsarcosine, purified through Ni–NTA chromatography only, single step purification and refolded into biologically active form. Our yield has been improved, and it is around 40 mg/litre. We identified and confirmed the purified protein by Western bolting as well as MALDI-ToF mass spectrometry. The expression and purification procedures reported in this study have provided a simple and efficient method to obtain pure CAVA protein used for further studies of its structure and function.

We have successfully cloned, expressed and purified CAVA protein in *E. coli*. We found that purified CAVA from their IBs under denatured condition is a good approach as we got high purity and concentration in single step. We are working on structure-based drug design (Hassan et al. 2007a, b, c; Shahbaaz et al. 2015) and discovery and CAVA is considered as a potential drug target, and some of its inhibitors may lead to the development of novel anti-obesity therapies (Arechederra et al. 2013; De Simone and Supuran 2007). Furthermore, structure analysis with docking and MD simulation will further guide to design a potent and selective inhibitor of CAV which will be validated experimentally.

## References

[CR1] Arechederra RL, Waheed A, Sly WS, Supuran CT, Minteer SD (2013). Effect of sulfonamides as carbonic anhydrase VA and VB inhibitors on mitochondrial metabolic energy conversion. Bioorg Med Chem.

[CR2] Boriack-Sjodin PA, Heck RW, Laipis PJ, Silverman DN, Christianson DW (1995). Structure determination of murine mitochondrial carbonic anhydrase V at 2.45-A resolution: implications for catalytic proton transfer and inhibitor design. Proc Natl Acad Sci USA.

[CR3] De Simone G, Supuran CT (2007). Antiobesity carbonic anhydrase inhibitors. Curr Top Med Chem.

[CR4] Dodgson SJ, Cherian K (1989). Mitochondrial carbonic anhydrase is involved in rat renal glucose synthesis. Am J Physiol.

[CR5] Dodgson SJ, Forster RE, Storey BT, Mela L (1980). Mitochondrial carbonic anhydrase. Proc Natl Acad Sci U S A.

[CR6] Ghandour MS, Parkkila AK, Parkkila S, Waheed A, Sly WS (2000). Mitochondrial carbonic anhydrase in the nervous system: expression in neuronal and glial cells. J Neurochem.

[CR7] Hassan MI, Kumar V, Kashav T, Alam N, Singh TP, Yadav S (2007). Proteomic approach for purification of seminal plasma proteins involved in tumor proliferation. J Sep Sci.

[CR8] Hassan MI, Kumar V, Singh TP, Yadav S (2007). Structural model of human PSA: a target for prostate cancer therapy. Chem Biol Drug Des.

[CR9] Hassan MI, Kumar V, Somvanshi RK, Dey S, Singh TP, Yadav S (2007). Structure-guided design of peptidic ligand for human prostate specific antigen. J Pept Sci.

[CR10] Hassan MI, Kumar V, Singh TP, Yadav S (2008). Purification and characterization of zinc alpha2-glycoprotein-prolactin inducible protein complex from human seminal plasma. J Sep Sci.

[CR11] Heck RW, Tanhauser SM, Manda R, Tu C, Laipis PJ, Silverman DN (1994). Catalytic properties of mouse carbonic anhydrase V. J Biol Chem.

[CR12] Ikai A, Tanaka S, Noda H (1978). Reactivation of kinetics of guanidine denatured bovine carbonic anhydrase B. Arch Biochem Biophys.

[CR13] Imtaiyaz Hassan M, Shajee B, Waheed A, Ahmad F, Sly WS (2013). Structure, function and applications of carbonic anhydrase isozymes. Bioorg Med Chem.

[CR14] Iqbal J, Al-Rashida M, Durdagi S, Alterio V, Di Fiore A (2015). Recent developments of carbonic anhydrase inhibitors as potential drugs. Biomed Res Int.

[CR15] Johnson WC (1999). Analyzing protein circular dichroism spectra for accurate secondary structures. Proteins.

[CR16] Kaul S, Ritschel WA (1988). Influence of obesity on sulfonamide disposition in Zucker rats. Eur J Drug Metab Pharmacokinet.

[CR17] Liang Z, Jonsson BH, Lindskog S (1993). Proton transfer in the catalytic mechanism of carbonic anhydrase. Effects of placing histidine residues at various positions in the active site of human isoenzyme II. Biochim Biophys Acta.

[CR18] McPhie P (2008). Concentration-independent estimation of protein secondary structure by circular dichroism: a comparison of methods. Anal Biochem.

[CR19] Nagao Y, Batanian JR, Clemente MF, Sly WS (1995). Genomic organization of the human gene (CA5) and pseudogene for mitochondrial carbonic anhydrase V and their localization to chromosomes 16q and 16p. Genomics.

[CR20] Parkkila AK, Scarim AL, Parkkila S, Waheed A, Corbett JA, Sly WS (1998). Expression of carbonic anhydrase V in pancreatic beta cells suggests role for mitochondrial carbonic anhydrase in insulin secretion. J Biol Chem.

[CR21] Perez-Iratxeta C, Andrade-Navarro MA (2008). K2D2: estimation of protein secondary structure from circular dichroism spectra. BMC Struct Biol.

[CR22] Picard F, Deshaies Y, Lalonde J, Samson P, Richard D (2000). Topiramate reduces energy and fat gains in lean (Fa/?) and obese (fa/fa) Zucker rats. Obes Res.

[CR23] Saarnio J, Parkkila S, Parkkila AK, Waheed A, Karttunen T, Sly WS (1999). Cell-specific expression of mitochondrial carbonic anhydrase in the human and rat gastrointestinal tract. J Histochem Cytochem.

[CR24] Shahbaaz M, Ahmad F, Hassan MI (2015). Structure-based function analysis of putative conserved proteins with isomerase activity from Haemophilus influenzae. 3 Biotech.

[CR25] Sreerama N, Woody RW (2000). Estimation of protein secondary structure from circular dichroism spectra: comparison of CONTIN, SELCON, and CDSSTR methods with an expanded reference set. Anal Biochem.

[CR26] Swenson ER (2014). Safety of carbonic anhydrase inhibitors. Expert Opin Drug Saf.

[CR27] van Karnebeek CD, Sly WS, Ross CJ, Salvarinova R, Yaplito-Lee J, Santra S, Shyr C, Horvath GA, Eydoux P, Lehman AM, Bernard V, Newlove T, Ukpeh H, Chakrapani A, Preece MA, Ball S, Pitt J, Vallance HD, Coulter-Mackie M, Nguyen H, Zhang LH, Bhavsar AP, Sinclair G, Waheed A, Wasserman WW, Stockler-Ipsiroglu S (2014). Mitochondrial carbonic anhydrase VA deficiency resulting from CA5A alterations presents with hyperammonemia in early childhood. Am J Hum Genet.

[CR28] Vitale RM, Pedone C, Amodeo P, Antel J, Wurl M, Scozzafava A, Supuran CT, De Simone G (2007). Molecular modeling study for the binding of zonisamide and topiramate to the human mitochondrial carbonic anhydrase isoform VA. Bioorg Med Chem.

